# Retraction: Inhibition of Rho-Kinase by Fasudil Suppresses Formation and Progression of Experimental Abdominal Aortic Aneurysms

**DOI:** 10.1371/journal.pone.0102393

**Published:** 2014-07-03

**Authors:** Chen Peng, Peng Gu, Jing Zhou, Jianhua Huang, Wei Wang

The authors retract this publication due to technical errors in the experiments reported.

During the process of summarizing data for another manuscript, the authors noticed that some of the data relate to experiments different from those described in the article. The samples for real-time PCR analysis and the aortic diameter measurements originate from the suprarenal aorta, and not the infrarenal aorta, which is the correct location for measurements in the porcine pancreatic elastase model.

Figure 1, Figure 2 and Figure 4 in the article are affected by the identified technical errors, and as a result, there is insufficient evidence to support the main conclusion in this paper. The authors therefore retract this publication. The authors deeply regret this circumstance and apologize to the community.
